# Supplementary material for: Low-Intensity Blood Flow Restriction Exercises Modulate Pain Sensitivity in Healthy Adults: A Systematic Review
Source: Healthcare (Basel). 2023 Mar 2;11(5):726. doi: 10.3390/healthcare11050726 (PMC10000465; doi:10.3390/healthcare11050726)
Supplement: Supplementary file 1 [file healthcare-11-00726-s001.zip › healthcare-2243576-supplementary.pdf]

Online Supplementary Material 1. Search Strategy

Databases: PubMed, CINAHL, EMBASE, PEDro, ScienceDirect, Cochrane Library<br>1. blood flow restriction<br>2. ischemic training<br>3. kaatsu<br>4. occlusion training<br>5. vascular occlusion<br>6. vascular restriction<br>7. 1 or 2 or 3 or 4 or 5 or 6<br>8. pain threshold<br>9. 7 and 8
MeSH Terms<br>1. BFR Therapy<br>2. BFR Therapies<br>3. Therapy, BFR<br>4. Blood Flow Restriction Training<br>5. Blood Flow Restriction Exercise<br>6. 1 or 2 or 3 or 4 or 5<br>7. Sensory threshold<br>8. 6 and 7
